# Locally harvested foods support serum 25-hydroxyvitamin D sufficiency in an indigenous population of Western Alaska

**DOI:** 10.3402/ijch.v73.22732

**Published:** 2014-03-20

**Authors:** Bret Luick, Andrea Bersamin, Judith S. Stern

**Affiliations:** 1 Center for Alaska Native Health Research, Institute of Arctic Biology, University of Alaska Fairbanks, Fairbanks, AK, USA; 2 Department of Nutrition, University of California, Davis, CA, USA

**Keywords:** Vitamin D, 25-hydroxyvitamin D, Alaska Natives, Traditional Foods, Seasonality, Dietary transition

## Abstract

**Background:**

Low serum vitamin D is associated with higher latitude, age, body fat percentage and low intake of fatty fish. Little documentation of vitamin D concentrations is available for Alaska Native populations.

**Objective:**

This study was undertaken to investigate serum 25-hydroxyvitamin D (25(OH)D) concentrations of the Yup'ik people of southwestern Alaska in relation to demographic and lifestyle variables, particularly with the use of locally harvested (local) foods.

**Design:**

Cross-sectional study.

**Methods:**

We estimated 25(OH)D, dietary vitamin D and calcium, percent of energy from local foods and demographic variables in 497 Yup'ik people (43% males) aged 14–92 residing in southwestern Alaska. Sampling was approximately equally divided between synthesizing and non-synthesizing seasons, although the preponderance of samples were drawn during months of increasing daylight.

**Results:**

Mean vitamin D intake was 15.1±20.2 µg/d, while local foods accounted for 22.9±17.1% of energy intake. The leading sources of vitamin D were local fish (90.1%) followed by market foods. Mean 25(OH)D concentration was 95.6±40.7 nmol/L. Participants in the upper 50th percentile of 25(OH)D concentration tended to be older, male, of lower body mass index, sampled during the synthesizing season, and among the upper 50th percentile of local food use.

**Conclusions:**

A shift away from locally harvested foods will likely increase the risk for serum 25(OH)D insufficiency in this population.

Vitamin D insufficiency is a widespread public health problem (1–4) that is more prevalent at higher latitudes because of reduced or nearly non-existent cutaneous (UV-B sunlight driven) pre-vitamin D synthesis for most of the year (5–7). Chronic vitamin D insufficiency results in rickets and osteoporosis and is associated with increased risk for a variety of conditions such as heart disease, stroke and type 2 diabetes (2, 8). Supplementation has been associated with reduction in overall mortality (9). The majority of serum 25(OH)D, the accepted standard for monitoring vitamin D status, is derived from cutaneous production, and at higher latitudes low serum 25(OH)D may be expected throughout much of the year (5, 10, 11). Dietary vitamin D can be an important contributor to serum 25(OH)D, although the vitamin D content of most Western diets is low (12, 13). By comparison, the diet of Yup'ik Eskimos of Western Alaska is marked by locally harvested (local) foods remarkably rich in vitamin D through consumption of fatty fish, fish roe and wild game (14, 15). While evidence indicates that vitamin D intake among Yup'ik Eskimos is currently adequate, we also know that local food use is in long-term decline, which may eventually compromise vitamin D intake, especially among youth (16). Yup'ik Eskimos are typically low consumers of dairy products, consistent with reported low lactose tolerance (17, 18) and low calcium intake (14, 15).

The combination of living at a relatively high latitude, along with few opportunities for sun exposure and high variability in fatty fish intakes and limited use of fortified dairy products, provided a unique opportunity to investigate the impact of changing dietary patterns on vitamin D status. The primary objectives of this study were to assess serum 25(OH)D concentrations among residents of Western Alaska and to identify demographic characteristics associated with 25(OH)D status. This research can identify the demographic and behavioural characteristics that, during this period of dietary change, predict risk of vitamin D insufficiency.

## Methods

### Study population and design

The study participants included 213 men and 284 women drawn from the Center for Alaska Native Health Study. This study was an interdisciplinary investigation of genetic, nutritional and behavioural risk factors for obesity and chronic diseases among Alaska Natives (19–21). The study protocol, using circum-annual convenience sampling, has been published previously (16). Briefly, residents of 6 remote communities and one town in the Yukon Kuskokwim River Delta, Alaska, were recruited to participate in the study via fliers, word of mouth and the locally popular Very High Frequency radio. A local field assistant was hired to assist in the recruitment process in each community. All male and non-pregnant female Yup'ik Eskimos ≥14 years old were eligible to participate. Fully informed written consent, and assent required for minors, was obtained from all participants. Height, weight, circumferences and skin-fold thickness were measured. Also, age, diet, socio-demographic characteristics, health and wellness were accounted for along with a blood sample. This paper presents findings from observations and samples collected between December 2003 and March 2005.

The University of Alaska at Fairbanks, the University of California at Davis and the Yukon Kuskokwim Health Corporation Institutional Review Boards approved the study protocol.

The Yukon Kuskokwim Delta is located in Western Alaska, approximately 60°N, and is home to approximately 16,500 Alaska Natives living predominantly in small remote communities (populations <500). The communities were accessed primarily by local air service companies as there is no road system. According to Census 2000 data, the median age in the census area is 25.3 years, compared to 35.3 in the US population. The majority of the population, particularly the younger generation, is bilingual, speaking both Yup'ik and English; 65.6% reported that a language other than English was spoken at home.

### Dietary assessment

Diet was assessed using interviewer administered 24-hour recall (24HR); a subsample of participants (n=293) also completed a 3-day diet record. Diet data were collected from each participant by certified interviewers using computer-assisted 24-hour dietary recall software (Nutrition Data System for Research [NDS-R] software version 4.06) (22). The NDS-R database contains estimation of vitamin D concentrations for all foods, based on the currently available evidence. Many Alaskan Native foods are found in the database. Foods missing from the database were either substituted for similar food items when appropriate or the food was added to the database by request. Since data from the 3-day food record and 24-hour recall were collected using different methodologies, nutrient estimations from each were standardized and combined to yield a single value using SPSS software (version 13.0, 2004, SPSS, Inc., Chicago, IL). The 4 standardized observations were then averaged and the single resulting value back-transformed to the original units on the basis of the mean of the 24-hour dietary recall. Unlike the 3-day diet records, the 24-hour dietary recall was available for all participants. Data from participants without a 3-day food record (n=204) were used as is. Local foods were defined as those foods harvested from the local environment and included berries, marine mammals, fish, game animals and wild greens (23). The contribution of locally harvested foods to mean energy intake was calculated on the basis of energy. Mixed foods were disaggregated, so only locally harvested ingredients were included in the calculation. Among locally consumed foods, foods of animal origin, comprising fish and fish roe (52.3%), seal oil (17.0%), game meat (14.2%), game fowl (10.5%), organ meats (2.0%), shellfish (1.2%) and animal fat (0.2%), accounted for 97.2% of the energy source. The remaining 2.8% of estimated locally harvested food energy intake comprised berries and wild greens (14).

### Biochemical assessment

Sample handling has been described previously (24). Briefly, blood specimens were collected by venipuncture after at least 8 hours of fasting and isolated red cell and serum fractions held at −20°C in the field until placed in −40°C long-term storage. Total vitamin D was analyzed by radioimmunoassay (Diasorin, Inc.).

### Data analyses

Statistical tests were performed with JMP 10 (SAS Institute, Cary, North Carolina, 2012). A 2-sided P-value <0.05 was considered statistically significant. Age categories were set to: 14–19, 20–49 and ≥50, consistent with serum 25(OH)D subsample stratification and earlier reporting (21). The seasonality of sampling date was defined as either synthesizing, corresponding to the semi-annual period with longer average day length (22 March–21 September), or non-synthesizing (22 September–21 March), the semi-annual period with shorter average day length.

Summary statistic P-values were calculated by *t*-tests. The Pearson Chi-square (χ^2^) test was used as a main effects test of the equality of distributions in cross-tabulations. The relationship between age, body mass index (BMI, 0–25, 25–30, ≥30), gender and season; intake of vitamin D, calcium and local foods; and 25(OH)D was evaluated by multiple regression. The intakes were split at the median value. Multicollinearity was estimated by the variance inflation factor, and while there are no formal cut-off criteria, we used a comparatively conservative value of 5 (25). No variables exceeded these criteria (the maximum was 1.5) and none were excluded. Since the current analyses sought to evaluate relationships between intake and a biological marker, the median values were used for cut-off points. Evaluation of the adequacy of intakes and 25(OH)D were based on current guidelines (26). The association between vitamin D and local food intake was evaluated with Spearman's rho statistic.

## Results

Demographic and clinical characteristics of participants are summarized by gender (Table I). Sampling occurred throughout the year and was closely divided between synthesizing (N=247) and non-synthesizing (N=250) seasons (χ^2^=0.018, P=0.89). More samples were drawn on days when sunlight was gaining (winter solstice–summer solstice, N=430) as opposed to decreasing (summer solstice–winter solstice, N=67, χ^2^=277, P<0.01) (data not shown). Age distribution, dietary intake of vitamin D and calcium, and 25(OH)D concentrations were similar between the genders. The study mean BMI was 27.7, with BMI significantly higher among women than men (ρ=0.41, P<0.01). Mean BMI exceeded the normal range cut-off point of 25 in both genders.

**Table I:** Age, dietary and clinical characteristics of 497 Alaska Native men and women^a^

Characteristic	*n*	Mean	Median	Men	Women	P
Sampling period	497			213	284	<0.01^b^
Synthesizing^c^	247			100	147	0.29
Non-synthesizing	250			113	137	
Age, years	497	39.0±17.4	38	39.4±18.0	38.7±16.9	0.68^d^
BMI (kg/m^2^)	497	27.7±6.12	26.8	25.9±4.48	29.1±6.79	<0.01
Vitamin D intake (µg/d)	497	15.1±20.2	8.6	18.0±25.8	12.9±14.4	<0.01
Calcium intake (mg/d)	497	416±293	340	470±340	376±244	<0.01
Energy from local foods (%)	497	22.9±17.1	18.9	23.0±17.6	22.8±16.7	0.9
Serum 25(OH)D (nmol/L)	497	95.6±40.7	89.9	101.1±44.4	91.6±37.2	0.01
Synthesizing	497	103.1±41.2	102.3	109.0±43.4	92.9±39.3	0.07
Non-synthesizing	497	88.3±38.9	82.4	93.9±44.5	83.6±33.0	0.04

BMI=body mass index.

a Data include counts, means±standard deviation and medians.

b Pearson χ^2^, like values. P=0.29 for sampling differences by sex by season.

c March 22–September 21.

d *t*-test, like values.

Dietary and serum Vitamin D was positively associated with consumption of local foods (ρ=0.52, P<0.01). The mean serum 25(OH)D across all participants was sufficient (≥50 nmol/L). Estimated calcium intakes largely failed to meet current dietary recommendations.

Frequency distributions for demographic, dietary and clinical characteristics, and season were calculated by quartile of 25(OH)D (Table II). The quartile cut-off points were 66.1, 89.9, 119.8 nmol/L, p≤0.05. Age, sampling period, vitamin D intake and energy from local foods showed significant inequalities in 25(OH)D distributions.

**Table II:** Frequency distribution by quartile of serum 25-OHD among 497 Alaska Native men and women of demographic, dietary and clinical characteristics, and season^a^

Variable	1	2	3	4	P^b^
Median [IQR]	52.4 [17.5–64.9]	77.4 [67.4–89.9]	104.8 [92.4–120]	147 [122–270]	
Age					<0.01
<20	57 (11.5)	28 (5.6)	8 (1.6)	3 (0.6)	
20–49	60 (12.1)	85 (17.1)	63 (12.7)	45 (9.1)	
≥50	7 (1.4)	17 (3.4)	56 (11.3)	68 (13.7)	
Gender					0.09
Men	77 (15.5)	72 (14.5)	79 (15.9)	56 (11.3)	
Women	47 (9.5)	58 (11.7)	48 (9.7)	60 (12.1)	
BMI					0.43
<25	55 (11.1)	47 (9.5)	38 (7.6)	45 (9.1)	
25–29.9	35 (7)	45 (9.1)	47 (9.5)	38 (7.6)	
≥30	34 (6.8)	38 (7.6)	42 (8.5)	33 (6.6)	
Sampling period					<0.01
Non-synthesizing^c^	79 (15.9)	73 (14.7)	54 (10.9)	44 (8.9)	
Synthesizing	45 (9.1)	57 (11.5)	73 (14.7)	72 (14.5)	
Vitamin D intake					<0.01
Lower	96 (19.3)	66 (13.3)	59 (11.9)	29 (5.8)	
Upper	28 (5.6)	64 (12.9)	68 (13.7)	87 (17.5)	
Calcium intake					0.11
Lower	53 (10.7)	65 (13.1)	62 (12.5)	68 (13.7)	
Upper	71 (14.3)	65 (13.1)	65 (13.1)	48 (9.7)	
Percent of energy from locally harvested foods					<0.01
Lower	84 (16.9)	66 (13.3)	60 (12.1)	38 (7.6)	
Upper	40 (8)	64 (12.9)	67 (13.5)	78 (15.7)	

BMI=body mass index.

a *n* (Total %).

b Pearson χ^2^.

c September 22–March 21.

Regression analysis was used to estimate the relationship between 25(OH)D concentration and categories of age, gender, BMI, sampling period, and vitamin D, calcium and local food intakes (Table III). The lowest referent state was assigned to the independent variable category associated with the lowest estimated 25(OH)D concentrations, corresponding to age category 14–19, women, BMI>30, winter sampling period, and the lower 50th percentile of vitamin D, calcium and traditional food intakes. With all predictor variables in their lowest referent state, serum 25(OH)D was estimated to be 39.7±5 nmol/L. The value of beta indicates the expected change in 25(OH)D from referent state with a change in the independent variable category. Therefore, a member of the 20- to 50-year-old age category was estimated to be 26.5 nmol/L higher in 25(OH)D, relative to 14- to 19-year-olds. Likewise, lower BMI, male gender, summer sampling period, and higher vitamin D and local food intakes were associated with a higher predicted 25(OH)D concentration. The standardized betas indicated that the largest predictor of 25(OH)D concentration was age, followed by sampling period and diet.

**Table III:** Multiple linear regression prediction of serum 25(OH)D among 497 Alaska Native men and women^a^

	Statistic		
	
Term	Beta	Std Beta	P
Intercept	39.7±5.02^b^		<0.01
Age 20–50 (year, referent 14–19)	26.5±4.2	0.26	<0.01
Age≥50 (year, referent 20–50)	32.0±3.5	0.36	<0.01
Men (referent women)	6.5±3.1	0.08	0.03
BMI 25–30 (referent≥30)	1.8±3.7	0.02	0.63
BMI <25 (referent 25–30)	9.1±3.6	0.11	0.01
Sampling period^c^ (referent non-synthesizing)	17.8±2.9	0.22	<0.01
Vitamin D, upper 50th % (referent lower 50th percentile)	7.2±3.3	0.09	0.03
Calcium, upper 50th % (referent lower 50th percentile)	0.1±3.1	0.00	0.99
Local food^d^, upper 50th % (referent lower 50th percentile)	10.3±3.6	0.13	<0.01

BMI=body mass index.

a nmol/L.

b Increased endogenous vitamin D synthesizing, March 22–September 21.

c Mean±SE.

d Locally harvested foods.

Dietary vitamin D was largely derived from locally harvested foods (Table IV). Fish, primarily locally harvested whitefish, salmon and herring, was the primary source of vitamin D. Milk was ranked 6th of all dietary vitamin D sources at 3% of the total (not shown). Also, milk was the main market source of vitamin D, followed by breakfast cereals (1.2%) and eggs (1.1%).

**Table IV:** Leading sources of vitamin D among Alaska Natives of Western Alaska^a^

	% of total
Food source	
Locally harvested	89.9
Market	10.1
Major food groupings^b^	
Meats	92.5
Dairy	3.75
Grains	2.1
Eggs	1.1
Beverages	0.2
Minor food groupings^b^	
Fish	90.1
Milk	3.2
Cereals	1.2
Eggs	1.1
Beef	1
Individual foods	
Whitefish	35.4
Salmon	26.8
Herring	13.1
Blackfish	4.1
Pike	3.1

a Up to 5 sources shown.

b Includes mixed dishes.

## Discussion

This analysis was conducted to assess the serum 25(OH)D status among individuals living in the Yukon Kuskokwim Delta of Alaska and to identify demographic and dietary covariates of 25(OH)D concentration. This relatively high latitude population practices a mixed subsistence lifestyle with a substantial dietary dependence on foods of marine origin. The main finding of this study was the relatively high mean serum vitamin D concentration, both during synthesizing and non-synthesizing seasons, despite the northerly latitude. The concentration of 25(OH)D was strongly correlated with age. Youths were more likely to have low serum 25(OH)D, and constitute an age group known to consume more market-based foods and less energy from vitamin D–rich local fish (14).

Westernization has been described in this population previously (16). We have previously demonstrated a strong association between the consumption of local foods and age and have shown that fish constituted the greater proportion of energy among those foods (14). Here, we show that diminished use of local foods, an estimate of acculturation, correlates with diminished 25(OH)D, and that 90.1% of vitamin D intake is attributable to fish. Furthermore, we have previously shown that Docosahexaenoic acid (DHA) and Eicosapentaenoic acid (EPA), 2 fatty acids abundant in foods of marine origin, are similarly distributed by age category in the red blood cell phospholipids of the same individuals (16). In addition, we have demonstrated the same age and diet association in red blood cell protein ^15^N enrichment, consistent with consumption of proteins from foods of a marine origin (24). The age effect has been attributed to changes in the efficiency of cutaneous production and time spent outdoors (11, 27). In this population, the fatty acid composition of red blood cell membranes is consistent with increased age-related consumption of vitamin D–rich local foods of marine origin (16, 24). Increased use of local foods and improved vitamin D status by age group is not unprecedented. Danish (28), Canadian Arctic (29–32), Icelandic (33) and Norwegian (34) populations exhibited increased serum 25(OH)D with age, also associated with fish consumption.

Nevertheless, low intake of vitamin D is of particular concern at higher latitudes as endogenous synthesis is constrained most of the year due to the limited exposure to sunlight of adequate intensity (5, 10, 35). Evidence suggests that sunlight-driven cutaneous production of vitamin D is typically the predominant source of serum vitamin D (1, 26, 34–37). Our analyses clearly show seasonality in 25(OH)D but with relatively high concentrations for the latitude (38). In the non-synthesizing season, we had 8.4% of cases classified as deficient (<50 nmol/L 25(OH)D, data not shown) as compared to 16–32% of a winter sample in Boston, MA (42°N) (38), despite residing at 60°N, some 18° farther north. In contrast, Gessner et al. (39) reported that 31% of 133 children aged between 6 and 23 months from 5 communities across Alaska of latitudes ranging from 58°N–71°N had 25(OH)D concentrations of less than 62.4 nmol/L. Mazess et al. (40) found a mean 25(OH)D of 41.4 nmol/L among 53 male and female Aleutian Islander (54°N) adults, which falls within the lowest referent category of the current report. Vitamin D deficiency has been reported for Southeast Alaska (approximately 58°N) based on medical record extraction where mean 25(OH)D concentration was 44.4±30.2 nmol/L among 55 non-diabetic patients (41). The non-synthesizing season mean 25(OH)D was above the highest NHANES III subgroup (42). However, this report is consistent with findings for indigenous adults residing in northern circumpolar regions where seasonality and local foods are important mediators of vitamin D intake (43) and circulating 25(OH)D concentration (27, 44–48). Pronounced seasonality of serum 25(OH)D has been demonstrated among 47 adult Caucasians residing in Fairbanks, Alaska, latitude 65°N, based on 12 months of longitudinal sampling and was shown to follow measured UV-B exposure, although intake appeared to be a more important factor than sunlight for determining year-round concentrations (49). The largely sufficient serum 25(OH)D values in the current report, particularly among elders, contrast sharply with other Alaska-based reports (40, 41, 42, 49) and are presumably due to increased adherence to a traditional lifestyle in the Yukon-Kuskokwim Delta of Western Alaska (50).

The temporal trend of 25(OH)D in any given participant was unknown, which limits the interpretation of seasonal effects, and longitudinal data collection should be considered. Determination of vitamin D and calcium in local foods as consumed, for instance solubilized in soups and contained in smoked fish skin, would improve estimated intakes.

In summary, vitamin D status was predominantly sufficient throughout the year and across the participants. Endogenously produced 25(OH)D was apparent during the synthesizing months, but the expected drop in serum 25(OH)D during other months seemed mitigated by dietary sources, which we attributed to the use of local foods, consistent with a traditional lifestyle.
